# Gene expression signature predicts human islet integrity and transplant functionality in diabetic mice

**DOI:** 10.1371/journal.pone.0185331

**Published:** 2017-10-02

**Authors:** Sunil M. Kurian, Kevin Ferreri, Chia-Hao Wang, Ivan Todorov, Ismail H. Al-Abdullah, Jeffrey Rawson, Yoko Mullen, Daniel R. Salomon, Fouad Kandeel

**Affiliations:** 1 Department of Molecular and Experimental Medicine, The Scripps Research Institute, La Jolla, California, United States of America; 2 Department of Translational Research and Cellular Therapeutics, Diabetes, and Metabolism Research Institute, City of Hope National Medical Center, Duarte, California, United States of America; Children's Hospital Boston, UNITED STATES

## Abstract

There is growing evidence that transplantation of cadaveric human islets is an effective therapy for type 1 diabetes. However, gauging the suitability of islet samples for clinical use remains a challenge. We hypothesized that islet quality is reflected in the expression of specific genes. Therefore, gene expression in 59 human islet preparations was analyzed and correlated with diabetes reversal after transplantation in diabetic mice. Analysis yielded 262 differentially expressed probesets, which together predict islet quality with 83% accuracy. Pathway analysis revealed that failing islet preparations activated inflammatory pathways, while functional islets showed increased regeneration pathway gene expression. Gene expression associated with apoptosis and oxygen consumption showed little overlap with each other or with the 262 probeset classifier, indicating that the three tests are measuring different aspects of islet cell biology. A subset of 36 probesets surpassed the predictive accuracy of the entire set for reversal of diabetes, and was further reduced by logistic regression to sets of 14 and 5 without losing accuracy. These genes were further validated with an independent cohort of 16 samples. We believe this limited number of gene classifiers in combination with other tests may provide complementary verification of islet quality prior to their clinical use.

## Introduction

The pathophysiology of Type 1 Diabetes Mellitus (T1DM) is the result of autoimmune destruction of insulin-producing beta cells in the pancreas. Several immunotherapy strategies are suggested in order to reduce the immune mediated destruction of the insulin producing cell [1,2]. In addition, a promising treatment paradigm for T1DM is replacement of the missing beta cells with islet cells isolated from allogeneic donor organs [3,4]. Successful islet transplantation has been shown to improve glycemic control, induce insulin independence or significantly reduce insulin requirements and, most importantly, provide several years of freedom from life-threatening hypoglycemic episodes [5–7]. Although concerted efforts from several groups have resulted in progress in the field over the last decade, transplantation outcomes have not been consistent between the various transplant centers [8]. In addition to problems with alloimmune rejection and residual auto-immunity directed against the islet graft, the ability of human islet isolation centers to consistently provide viable and functional islet cells varies widely within and especially between transplant centers [8,9]. This is confounded by the lack of robust, reproducible and standardized methods for gauging the suitability of specific islet preparations for clinical transplantation [10–12].

Consequently, a major effort in the field has been the development of methods for evaluating islets prior to clinical transplantation which are predictive of outcomes in the patients. Currently, the best evidence of islet function is reversal of diabetes by transplantation of human islets into diabetic immunocompromised mice [13]. However, the assay requires several weeks to obtain results and is therefore not suitable for assessment of the cells prior to transplantation, which typically occurs within three days post-isolation. As a result, research efforts have focused on the identification of surrogate parameters that are predictive of islet graft function and which can be evaluated within the relatively short time between islet isolation and infusion into the patient.

Our group has investigated the use of percent beta cell apoptosis (BAP) and glucose-responsive oxygen consumption rates (OCR) as predictors of islet graft function. Each of these approaches independently predicts reversal of diabetes in mice with reasonable accuracy (0.856 for BAP [14] and 0.793 for OCR [15]). Furthermore, these methods are rapid enough to obtain results prior to clinical use of the islet preparations. We also demonstrated that OCR provides identical results independent of the institute performing the assay. However, these widely used *in vitro* approaches focus solely on the immediate integrity of the islet preparation without regard to potential for *in vivo* islet function or graft-host interaction, elements more likely to be important for long-term efficacy following transplantation.

In considering the factors that make an islet preparation “*good*” for clinical use, we speculated that both the function of the islet preparation and the interaction with the recipient would be governed by the expression of specific islet genes. Therefore *good* islet preparations would have a distinctive “gene signature”. To test this hypothesis, whole genome RNA expression analysis using microarrays was performed on 59 human islet preparations in parallel with assessment of islet function by transplantation into diabetic mice. Using this approach, a set of 262 microarray probesets representing 199 human genes was associated with the ability of islets to reverse diabetes in mice. These probesets were able to predict the outcome of transplantation studies with an accuracy of over 83%, suggesting that a “gene signature” could be associated with islet quality.

Importantly, the gene classifiers were functionally associated with islet biology and were predominantly associated with inflammation and repair mechanisms rather than metabolic function. Interestingly, the gene signature showed little overlap with gene expression profiles associated with our other measures of islet quality, BAP and OCR, suggesting these islet quality tests measure different aspects of islet biology. Finally, we demonstrate that the microarray-based gene signature assessment is readily adaptable to rapid evaluation of islet preparations using a PCR based methodology. In summary, our data demonstrate the feasibility of using islet gene expression as a metric for functional islet quality assessment in the context of clinical cell therapy programs.

## Results

### Islet gene signature correlated with reversal of diabetes

To identify a gene signature associated with islet quality, each islet preparation was assigned to one of two classes based on their ability to reverse diabetes, namely *good* islets, which resulted in reversal of diabetes after transplantation into diabetic mice, and *bad* islets, those which failed to reverse diabetes (see Materials and methods for criteria). To minimize bias in the analysis, the 59 samples were randomly assigned three times into two groups, a training set (Group 1) and a validation set (Group 2), while maintaining approximately equal numbers of good and bad samples in each group. In each iteration Group 1 was used to identify microarray probesets representing individual genes that were associated with either good or bad islet preparations, then Group 2 was used to test each of the resultant probesets for the ability to correctly predict the category (*good* or *bad*) of each islet preparation (see Materials and methods for a detailed description). Probesets that had 100% cross-validation efficiency (%CV; i.e. effectiveness at classifying the samples correctly) were collected as a Predictor Classifiers list. The combined Predictor Classifiers from the three randomizations yielded a total of 262 unique probeset classifiers for islet quality representing 199 genes that had 100% predictive accuracy (Table 1). The data showed that 135 of the 262 probesets (51.5%) exhibited higher expression levels in *bad* islet preparations, and 127 probesets had higher expression in *good* islet preparations.

**Table 1:** Probeset classifiers for reversal of diabetes arranged by p-value. The 36 classifier subset is highlighted in BOLD. P-values are the Parametric P-values obtained during the analyses of all the microarray datasets. Fold-change is the ratio of average expression (intensity level) in the Bad samples divided by average expression in the Good samples. Probes are the identification numbers of the Affymetrix U133 Plus 2.0 GeneChip probesets.

#	P-value	Fold-change	Symbol	Probe	#	P-value	Fold-change	Symbol	Probe
**1**	**0.0001**	**1.54**	**MYOF**	**211864_s_at**	132	0.0025	0.68	ABHD6	45288_at
**2**	**0.0002**	**0.66**	**MAPT**	**203929_s_at**	133	0.0025	0.73	UCHL1	201387_s_at
3	0.0002	0.74	CCDC108	239508_x_at	134	0.0026	1.3	FBLIM1	1555480_a_at
4	0.0002	1.24	SLC44A1	224595_at	135	0.0026	1.23	RASSF9	210335_at
5	0.0002	1.63	--	235144_at	**136**	**0.0026**	**0.78**	**TSHZ3**	**223393_s_at**
6	0.0002	0.77	USP30	227572_at	**137**	**0.0027**	**0.76**	**DENND5B**	**228551_at**
7	0.0002	1.46	--	232478_at	138	0.0027	1.42	--	236114_at
**8**	**0.0003**	**1.43**	**NOTCH2**	**212377_s_at**	139	0.0027	1.34	PYGL	202990_at
9	0.0003	0.7	N4BP2L2	214748_at	140	0.0027	0.7	--	1558170_at
10	0.0003	0.81	--	227547_at	141	0.0027	0.85	ZYG11B	225338_at
11	0.0003	0.66	TBC1D4	203386_at	142	0.0027	1.53	ZFP36L1	211962_s_at
**12**	**0.0003**	**2.03**	**ITGB6**	**208083_s_at**	143	0.0028	1.26	PGM2L1	235149_at
**13**	**0.0004**	**0.75**	**RNF187**	**229207_x_at**	144	0.0028	1.47	LPAR6	218589_at
**14**	**0.0004**	**0.78**	**TSHZ1**	**223282_at**	145	0.0028	0.68	SRD5A1	204675_at
15	0.0004	0.64	FBXL14	1553683_s_at	146	0.0028	0.82	CSRP2BP	225432_s_at
16	0.0004	0.8	ARPP19	221482_s_at	147	0.0029	1.64	OSMR	205729_at
17	0.0005	1.47	--	216565_x_at	148	0.0029	1.25	--	237310_at
**18**	**0.0005**	**0.76**	**ZC3H6**	**227809_at**	149	0.0029	0.8	FAM55C	243606_at
19	0.0006	1.29	GRHL2	219388_at	150	0.0029	1.17	BAT1	200041_s_at
**20**	**0.0006**	**1.43**	**FAM186A**	**216595_at**	151	0.0030	0.85	FLJ35390	1569090_x_at
21	0.0006	1.46	CASP4	209310_s_at	152	0.0030	0.8	LOC284440	1555363_s_at
22	0.0006	0.77	NEBL	203961_at	153	0.0030	0.74	FLJ35390	1569089_a_at
23	0.0006	0.75	LONRF2	225996_at	154	0.0030	1.42	FOSL2	225262_at
24	0.0006	0.75	--	242651_at	155	0.0030	0.77	CADPS	204814_at
25	0.0007	1.25	FRMD4A	208476_s_at	156	0.0030	0.8	C15orf61	229742_at
26	0.0007	0.8	SALL2	213283_s_at	157	0.0030	1.52	OPN3	224392_s_at
27	0.0007	0.85	NGRN	217722_s_at	158	**0.0030**	**1.35**	**SEPT9**	**208657_s_at**
28	0.0007	1.28	EXT1	201995_at	159	0.0030	1.31	KIAA1949	224927_at
29	0.0007	0.82	--	1569478_s_at	160	0.0031	0.8	USP2	229337_at
30	0.0008	1.43	CPM	241765_at	161	0.0031	0.83	FAM111B	1557128_at
**31**	**0.0008**	**2.17**	**ITGB6**	**226535_at**	**162**	**0.0031**	**1.32**	**NOTCH2**	**210756_s_at**
32	0.0008	0.57	VAT1L	226415_at	163	0.0031	0.81	ATP1B2	204311_at
33	0.0008	0.66	HADH	201035_s_at	164	0.0031	0.79	GPRIN1	227975_at
34	0.0008	0.67	CYP2U1	226393_at	165	0.0031	0.7	SGSM1	230287_at
**35**	**0.0009**	**0.58**	**--**	**236660_at**	166	0.0031	0.76	DLEU1	205677_s_at
**36**	**0.0010**	**0.61**	**KCNMA1**	**228414_at**	167	0.0031	1.2	ABCC1	202805_s_at
37	0.0011	1.35	DSC2	204751_x_at	**168**	**0.0031**	**1.72**	**PMEPA1**	**222450_at**
**38**	**0.0011**	**1.72**	**PMEPA1**	**222449_at**	169	0.0032	1.43	--	214803_at
39	0.0011	1.57	CD44	217523_at	170	0.0032	1.51	PDGFC	222719_s_at
40	0.0011	1.28	PEX11A	205161_s_at	171	0.0032	1.42	FLRT3	222853_at
41	0.0011	1.74	--	232277_at	172	0.0033	1.72	TGFB2	228121_at
**42**	**0.0012**	**1.26**	**EHD4**	**209536_s_at**	173	0.0033	0.55	--	1559111_a_at
43	0.0012	1.32	TYMP	217497_at	174	0.0033	0.72	GPR44	206361_at
44	0.0012	1.37	--	232174_at	175	0.0033	1.39	TPBG	203476_at
45	0.0012	0.78	DENND5B	215058_at	176	0.0033	0.8	DGKE	238694_at
46	0.0012	0.63	--	230932_at	177	0.0033	0.7	HADH	211569_s_at
47	0.0013	1.38	PLSCR1	202430_s_at	178	0.0033	0.67	TBC1D4	203387_s_at
48	0.0013	0.79	HIPK2	225368_at	179	0.0033	0.81	GABRB3	227830_at
49	0.0013	0.7	MAPT	225379_at	180	0.0034	0.76	RRAGD	221523_s_at
50	0.0013	1.23	ETS2	241193_at	181	0.0034	0.7	--	1559110_at
51	0.0014	1.29	--	226756_at	182	0.0035	0.85	--	239122_at
**52**	**0.0014**	**1.32**	**MPZL2**	**203779_s_at**	183	0.0035	1.51	NRP1	212298_at
53	0.0014	1.27	--	227184_at	184	0.0035	1.27	--	227167_s_at
54	0.0014	1.43	IFI44	214453_s_at	185	0.0035	1.89	C3	217767_at
55	0.0015	1.17	RYK	214172_x_at	186	0.0035	0.63	PPM1E	236302_at
56	0.0015	0.69	G12	231296_at	**187**	**0.0035**	**1.44**	**NOTCH2**	**202443_x_at**
**57**	**0.0015**	**0.76**	**STXBP5L**	**240236_at**	188	0.0035	0.82	CCDC109B	218802_at
58	0.0015	0.82	ZNF791	1553703_at	189	0.0035	1.74	SERPI3	202376_at
59	0.0015	0.68	RAB39B	230075_at	190	0.0035	1.38	CSRP2	207030_s_at
60	0.0015	1.34	LITAF	200706_s_at	191	0.0036	0.91	C1orf43	223034_s_at
61	0.0016	0.81	ZNF791	1553704_x_at	192	0.0036	1.35	--	232925_at
**62**	**0.0016**	**0.77**	**--**	**231331_at**	**193**	**0.0036**	**0.85**	**LOC401913**	**244176_at**
63	0.0016	1.23	ARHGAP18	225171_at	**194**	**0.0036**	**1.36**	**PLSCR1**	**202446_s_at**
64	0.0016	1.38	FCER1G	204232_at	195	0.0036	1.29	--	243252_at
65	0.0016	0.72	C6orf174	233050_at	196	0.0036	0.73	KIF5C	203129_s_at
66	0.0016	0.72	NKX6-1	221366_at	197	0.0036	1.54	ANGPTL4	221009_s_at
67	0.0016	0.7	ASCL2	229215_at	198	0.0037	0.85	FANCD2	1568889_at
68	0.0016	1.44	YAP1	224895_at	199	0.0037	1.7	LGALS1	201105_at
**69**	**0.0016**	**0.74**	**PTPN3**	**227944_at**	200	0.0037	1.31	FAM102B	226568_at
70	0.0017	0.83	PNMA1	218224_at	201	0.0037	0.76	SOBP	218974_at
71	0.0017	1.63	FGG	226621_at	202	0.0037	1.39	LTF	202018_s_at
72	0.0017	1.34	NFIB	211467_s_at	203	0.0037	0.81	ANKH	223092_at
73	0.0017	0.64	CYP2U1	226402_at	204	0.0037	0.79	ROBO2	240425_x_at
74	0.0017	0.82	CGRRF1	204605_at	205	0.0037	0.62	--	231040_at
75	0.0017	1.23	ELF1	212420_at	206	0.0038	0.67	PPM1H	212686_at
**76**	**0.0017**	**1.55**	**--**	**224999_at**	207	0.0038	0.72	CASR	211384_s_at
77	0.0018	1.44	IFITM3	212203_x_at	208	0.0038	1.2	CNN2	201605_x_at
78	0.0018	1.4	B4GALT1	238987_at	**209**	**0.0038**	**0.67**	**PKIB**	**223551_at**
79	0.0018	1.35	SPRED1	226837_at	210	0.0038	0.65	RAB39B	238695_s_at
80	0.0018	0.82	SVIP	226278_at	211	0.0038	0.73	WFS1	202908_at
**81**	**0.0018**	**1.47**	**IFITM2**	**201315_x_at**	212	0.0039	0.62	NR0B1	206645_s_at
**82**	**0.0019**	**1.4**	**CARD6**	**224414_s_at**	213	0.0039	1.31	FLT3	206674_at
83	0.0019	0.74	--	210674_s_at	214	0.0039	0.85	LOC100132767	1569522_at
84	0.0019	1.38	CNN3	201445_at	215	0.0039	1.37	NFIB	209290_s_at
85	0.0019	1.44	FST	226847_at	216	0.0040	1.35	PAG1	227354_at
86	0.0019	0.73	UBE2QL1	226612_at	217	0.0040	1.38	TANC1	225308_s_at
87	0.0019	0.75	PNMA2	209598_at	218	0.0040	0.87	TERF2IP	201174_s_at
88	0.0019	0.87	--	244505_at	219	0.0041	1.89	CCL2	216598_s_at
**89**	**0.0019**	**0.81**	**--**	**230039_at**	220	0.0041	0.72	SRD5A1	211056_s_at
90	0.0020	0.75	SEPT3	223362_s_at	221	0.0041	1.39	LAPTM5	201721_s_at
91	0.0020	1.47	EFEMP1	201842_s_at	222	0.0041	1.37	MARCKS	225897_at
92	0.0020	1.33	--	1557543_at	223	0.0041	0.81	ELMO2	55692_at
93	0.0020	0.78	SIPA1L2	225056_at	**224**	**0.0042**	**0.83**	**--**	**240455_at**
94	0.0021	1.55	PLIN2	209122_at	**225**	**0.0042**	**0.74**	**MNX1**	**214614_at**
95	0.0021	1.26	ITGB1	1553678_a_at	226	0.0042	0.85	DJC24	213853_at
96	0.0021	0.74	PTCH1	209815_at	227	0.0042	0.75	NEBL	203962_s_at
97	0.0021	1.24	SLC43A3	213113_s_at	228	0.0042	0.78	ZNF91	206059_at
98	0.0021	1.38	CSRP2	211126_s_at	229	0.0042	1.36	MAP3K13	1562440_at
99	0.0021	1.23	BAIAP2L1	222675_s_at	230	0.0044	1.41	REST	212920_at
100	0.0021	1.21	--	238973_s_at	231	0.0044	1.49	ABCC3	208161_s_at
101	0.0021	0.62	INSM1	206502_s_at	232	0.0044	0.85	LOC84989	1552665_at
102	0.0021	0.78	STXBP1	202260_s_at	233	0.0044	0.75	DOCK3	213482_at
103	0.0021	1.73	TLR3	206271_at	**234**	**0.0045**	**1.42**	**RND3**	**212724_at**
104	0.0021	0.72	SLC2A13	227176_at	235	0.0045	0.82	CLIP3	212358_at
105	0.0021	0.83	SLC7A14	232904_at	236	0.0045	1.46	--	239519_at
106	0.0021	1.57	CPM	235706_at	237	0.0045	0.74	BTBD3	202946_s_at
107	0.0022	1.44	NFIB	213033_s_at	238	0.0045	1.27	NMI	203964_at
108	0.0022	1.5	C1RL	218983_at	239	0.0046	0.79	FAM117A	221249_s_at
109	0.0022	0.72	--	1556160_a_at	240	0.0046	0.7	EFHD1	209343_at
110	0.0022	0.77	SYBU	218692_at	241	0.0046	0.7	KIAA2022	244370_at
111	0.0023	0.78	--	226964_at	242	0.0046	1.46	RBPMS	207836_s_at
**112**	**0.0023**	**1.73**	**PMEPA1**	**217875_s_at**	243	0.0046	1.29	GALNT2	217788_s_at
113	0.0023	1.49	GBP1	202269_x_at	244	0.0046	1.51	ABCC3	209641_s_at
114	0.0023	1.4	ONECUT1	210745_at	245	0.0047	1.22	--	1560230_at
115	0.0023	0.82	CRMP1	202517_at	246	0.0047	0.81	RNF180	242985_x_at
**116**	**0.0023**	**1.35**	**MAP3K5**	**203836_s_at**	247	0.0047	0.81	TMCC2	213096_at
117	0.0024	1.21	TGFBR1	236561_at	248	0.0047	1.24	RHOV	241990_at
118	0.0024	1.32	TNFRSF10B	209295_at	249	0.0048	1.42	F3	204363_at
119	0.0024	0.88	PAX1	1553492_a_at	250	0.0048	0.65	OLFM1	213131_at
120	0.0024	0.75	FOXE1	206912_at	251	0.0048	1.24	ANK3	207950_s_at
121	0.0024	0.71	MAPT	203930_s_at	252	0.0048	0.65	DACH2	239738_at
122	0.0024	0.82	FOXA2	40284_at	253	0.0048	1.24	--	220990_s_at
123	0.0024	0.86	ASB1	212818_s_at	**254**	**0.0048**	**1.29**	**FRMD4A**	**225163_at**
124	0.0024	1.38	IVNS1ABP	206245_s_at	255	0.0049	0.71	NKX2-2	206915_at
125	0.0024	1.52	NTN4	223315_at	256	0.0049	0.82	MAP1B	226084_at
126	0.0024	0.86	TPRG1L	224871_at	257	0.0049	0.77	BSN	204586_at
127	0.0024	0.62	LOC283454	229552_at	258	0.0049	1.36	SEMA4B	234725_s_at
**128**	**0.0025**	**1.42**	**ITGB6**	**208084_at**	259	0.0049	0.88	HERPUD2	222751_at
129	0.0025	1.45	FOSL2	218880_at	260	0.0049	1.34	LOC100128501	229296_at
130	0.0025	0.77	ARPP19	214553_s_at	261	0.0050	0.77	--	225685_at
131	0.0025	1.25	MYO1B	212365_at	262	0.0050	0.78	ICA1L	230454_at

**Fig 1:**
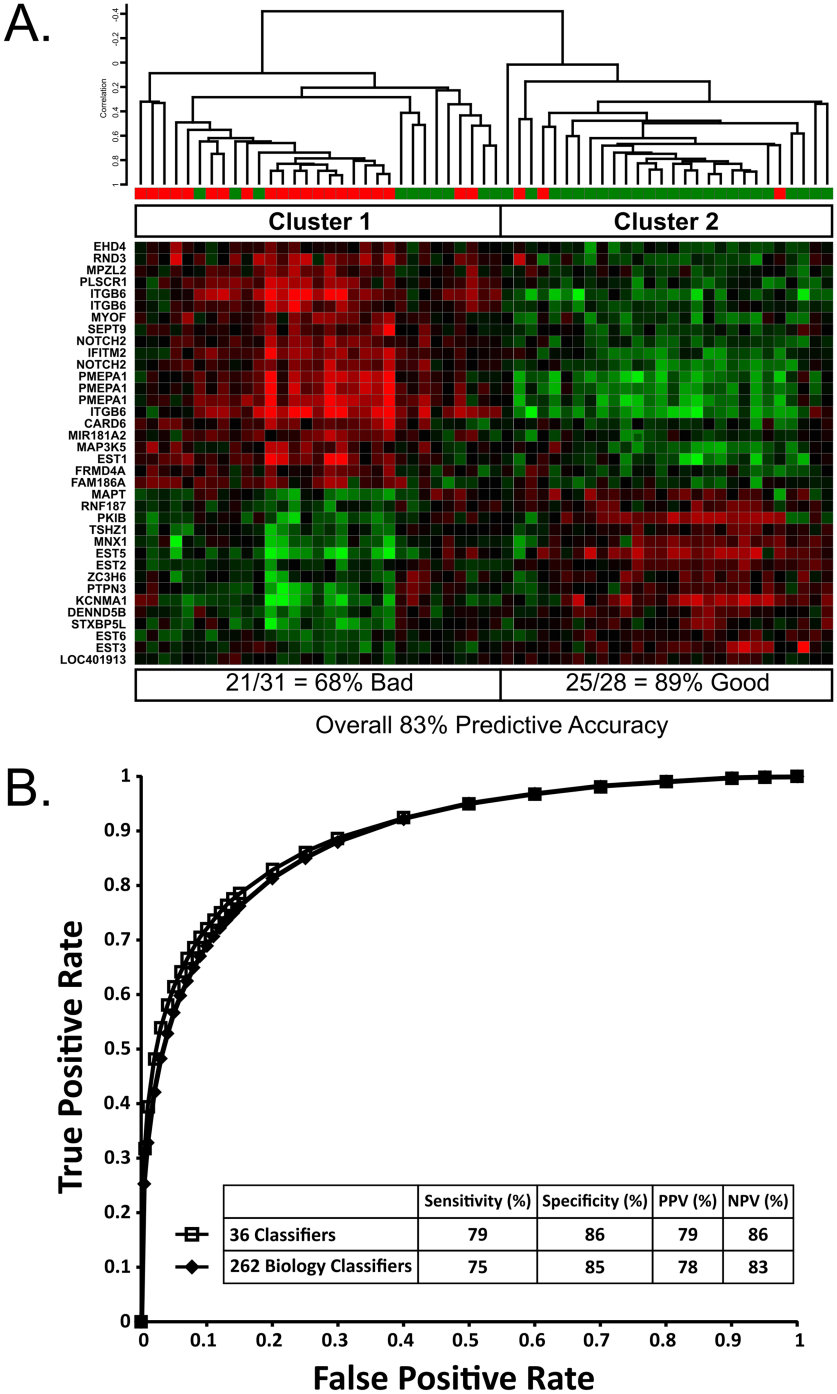
The 262 and 36 probeset lists predict islet quality. (A) 59 human islet preparations were clustered using the 262 probeset classifier. Eighty-nine percent (25 of 28 samples) of the *good* islets (green) clustered within the same quality class, and 68% (21 of 31 samples) of *bad* preparations (red) clustered together. The overall predictive accuracy of this classifier set was 83%. The heat map depicts expression level of the 36 probesets in each sample; *Red*: probeset with higher expression in *bad* islets; *Green*: probeset with higher expression in *good* islets; the intensity corresponds to the fold-difference in gene expression. (B) The 262 classifier set (diamonds) and the 36 classifier (squares) were analyzed by ROC curve analysis for their ability to discriminate between the *good* and *bad* classes of islet preparations. Both classifiers perform better at identifying poor islet preparations (NPV) than effective preparations (PPV).

The predictive value of the combined 262 probeset was then tested by supervised clustering of the 59 islet preparations. Cluster analysis generated two distinct clusters (Fig 1A) with the majority of *bad* islet preparations in Cluster 1 and majority of *good* preparations in Cluster 2. Only three *bad* islet preparations were misclassified resulting in an 89% predictive accuracy for the *good* preparations. However, ten of the total 36 *good* samples were misclassified as *bad* (27%). Notably, seven of these ten misclassified *good* preparations were in a small sub-cluster adjacent to another sub-cluster containing 2 of the 3 misclassified *bad* preparations, suggesting the possibility that these smaller clusters on both sides of the line of separation represent a class of intermediate quality islets. Overall however, these data demonstrate that using the consensus set of 262 probesets as a predictor would result in the transplantation of very few *bad* islet preparations.

Nevertheless, expression analysis of such a large number of genes may be difficult to implement in a clinical transplantation program on a routine basis, therefore we investigated ways to reduce the number of classifiers without losing predictive power. The Predictor Classifier lists from the three randomizations were compared and it was observed that they shared 36 “core” classifiers. The 36 classifiers clustered the samples into two groups with a distinct gene expression pattern for each class of samples (Fig 1A). The clusters predicted by the 36 probeset list were identical to the groups predicted by the 262 probeset list and also had an overall predictive value of 83%. Receiver operating characteristic analysis showed that the curves of two classifier sets (Fig 1B) were nearly identical, indicating that the reduced set of classifiers had equivalent predictive accuracy to the larger set, and that it correctly identified 79% of the *good* islet preparations (PPV; positive predictive value) and 86% of the *bad* preparations (NPV; negative predictive value).

Further analysis of the 36 probeset classifier was done using ANOVA gene expression model (Partek Genomics Suite) to classify each sample in each of six randomized sample groups using the probesets to determine its ability to predict islet function *in vivo*. The correct classification (*good* or *bad*) of each of the six random groups of samples ranged from 82% to 90.5% with an average of 85.3% correct prediction of the outcome in mice. In summary, both the 262 probeset classifier and the reduced list of 36 probesets, representing just 25 genes, predicted post-transplantation islet function with comparable and high accuracy.

### Refinement of predictor list by logistic regression

**Fig 2:**
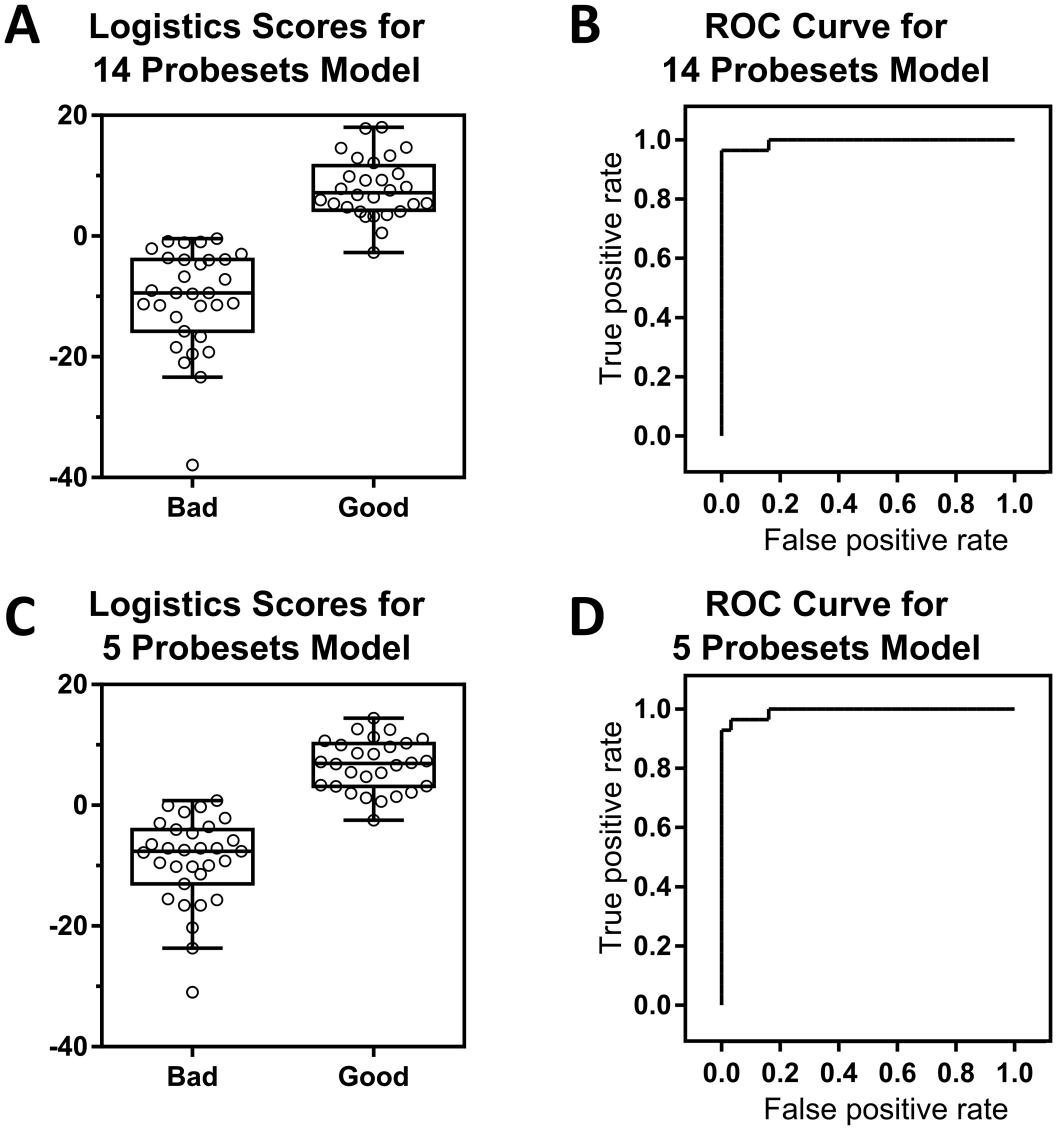
Reduction to 14 and 5 probeset lists by logistic regression. (A) Boxplot and (B) ROC curve for the fourteen probesets model. The fourteen probesets are EST2, KCNMA1, EST5, PKIB, EHD4, SEPT9, MIR181A2, RND3, PMEPA1, IFITM2, CARD6, MNX1, RNF187, MAPT. The model achieves a 0.96 true positive rate and a zero false positive rate at score threshold value of 0.5. (C) Boxplot and (D) ROC curve for the five probesets model. The five probesets are EST2, KCNMA1, RND3, PMEPA1, CARD6. The model achieves a 0.93 true positive rate and a zero false positive rate at score threshold value of 1.2.

To further assess redundancy in the 36 probeset above that is necessary for the successful classification of islets into good and bad preparations, logistic regression analyses were conducted. The results showed considerable redundancy even among the 36 probesets, with fourteen probesets in the initial full model (EST2, KCNMA1, EST5, PKIB, EHD4, SEPT9, MIR181A2, RND3, PMEPA1, IFITM2, CARD6, MNX1, RNF187, MAPT) showing excellent separation between good and bad islets, with a maximum 0.96 true positive rate and a zero false positive rate (Fig 2A and 2B). Interestingly, using further step-wise model simplification, the number of gene probes can be reduced down to five (EST2, KCNMA1, RND3, PMEPA1, CARD6) while maintaining a maximum true positive rate of 0.93 and a false positive rate of zero (Fig 2C and 2D).

### Classifier gene function

**Fig 3:**
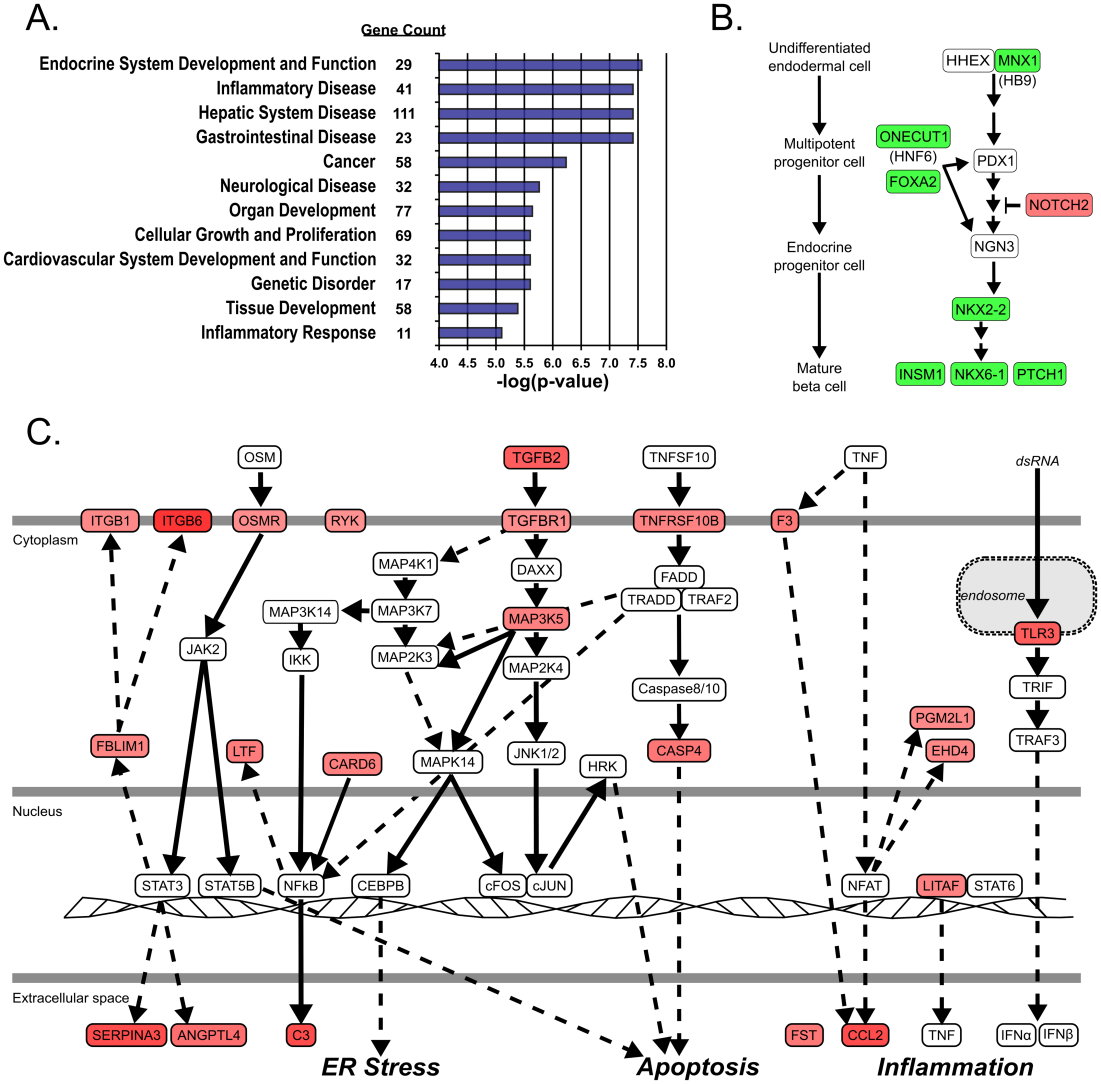
Functional pathways of the 262 classifier set. (A) Ingenuity Pathway Analysis was used to group the classifiers for reversal of diabetes into functional pathways. The twelve most significant functional pathways are listed in order of–log(p-value) with the most significant pathway (Endocrine System Development; p = 2.60 x 10^−9^) at the top. The number of probesets associated with each pathway is also listed. (B) Pancreatic endocrine cell development and regeneration pathway showing genes identified in the 262 classifier list (colored). (C) The network of inflammatory and immune related molecules predominantly expressed in non-functional islets prior to transplantation. Green means higher expression in *good* preparations, Red means higher expression in *bad* preparations, and the intensity corresponds to the fold-difference in gene expression. The pathway figures are adapted from Ingenuity Pathway analysis and the KEGG pathway database.

Examination of the 262 probesets (Table 1) revealed that approximately half (135 of 262) were more highly expressed in *bad* islet preparations while the other half were higher in *good* islet preparations, suggesting that the difference observed in *in vivo* function was not solely due to up-regulation of deleterious molecules, but also to the preservation or up-regulation of beneficial molecules. Further investigation of gene function by Ingenuity Pathway Analysis revealed that two apparent processes were competing in the islets to affect their *in vivo* function (Fig 3A). The most significant functional network was Endocrine System Development, which along with certain less significant networks (Cancer, Organ Development, Cellular Growth and Proliferation, Cardiovascular System Development, and Tissue Development), indicates an increase in tissue repair mechanisms such as cell proliferation and cell differentiation. Of the genes associated with endocrine development, 73% exhibit higher expression in *good* islet preparations, and together these genes form a pathway associated with beta cell development (Fig 3B). Increased expression of cell growth and differentiation pathways implies that ongoing islet repair is associated with better *in vivo* function.

The second most significant functional network was Inflammatory Disease (Fig 3A). Along with other networks (Hepatic System Disease, Gastrointestinal Disease, Neurologic Disease, and Inflammatory Response), these classifiers form an interconnected network of molecules associated with the innate immune system and the inflammatory response (Fig 3C). Importantly, 69% of the genes in Inflammatory Disease have higher expression in *bad* islets, suggesting that beta cells may participate in their own dysfunction by up-regulation of pro-inflammatory molecules even prior to transplantation. Some of these, such as CCL2 (also known as MCP1), have been previously reported to have a negative effect on islet function [16–19]. However, most of the genes in these pathways have not been studied in relation to islet biology or diabetes.

### Gene signatures associated with oxygen consumption rates and apoptosis

We previously reported that both the percentage of apoptotic beta cells (BAP) [14] and glucose-responsive oxygen consumption rates (OCR) [15] individually provided reasonable predictive accuracies of subsequent islet graft function of 0.86 (95% confidence interval: 0.75–0.96) and 0.79 (95% confidence interval: 0.61–0.97), respectively. OCR and BAP results represent different aspects of the islet preparation, with OCR reflecting the metabolic responsiveness of the islets and BAP the viability of the beta cell population. To determine the extent to which genes responsible for these characteristics were shared with each other and with the 262 probeset classifier, islet gene expression data was analyzed to identify genes associated with OCR and BAP results. To obtain an accurate gene expression profile for these characteristics using the current microarray datasets, transplantation results of current samples were used to set the thresholds of each method. For this sample set, the thresholds for OCR *good* and *bad* islets were OCR >0.191 and <0.085 nmol O_2_/min/100 islets, respectively. Similarly, *good* and *bad* islets had BAP <1.91% and >4.30%, respectively.

**Fig 4:**
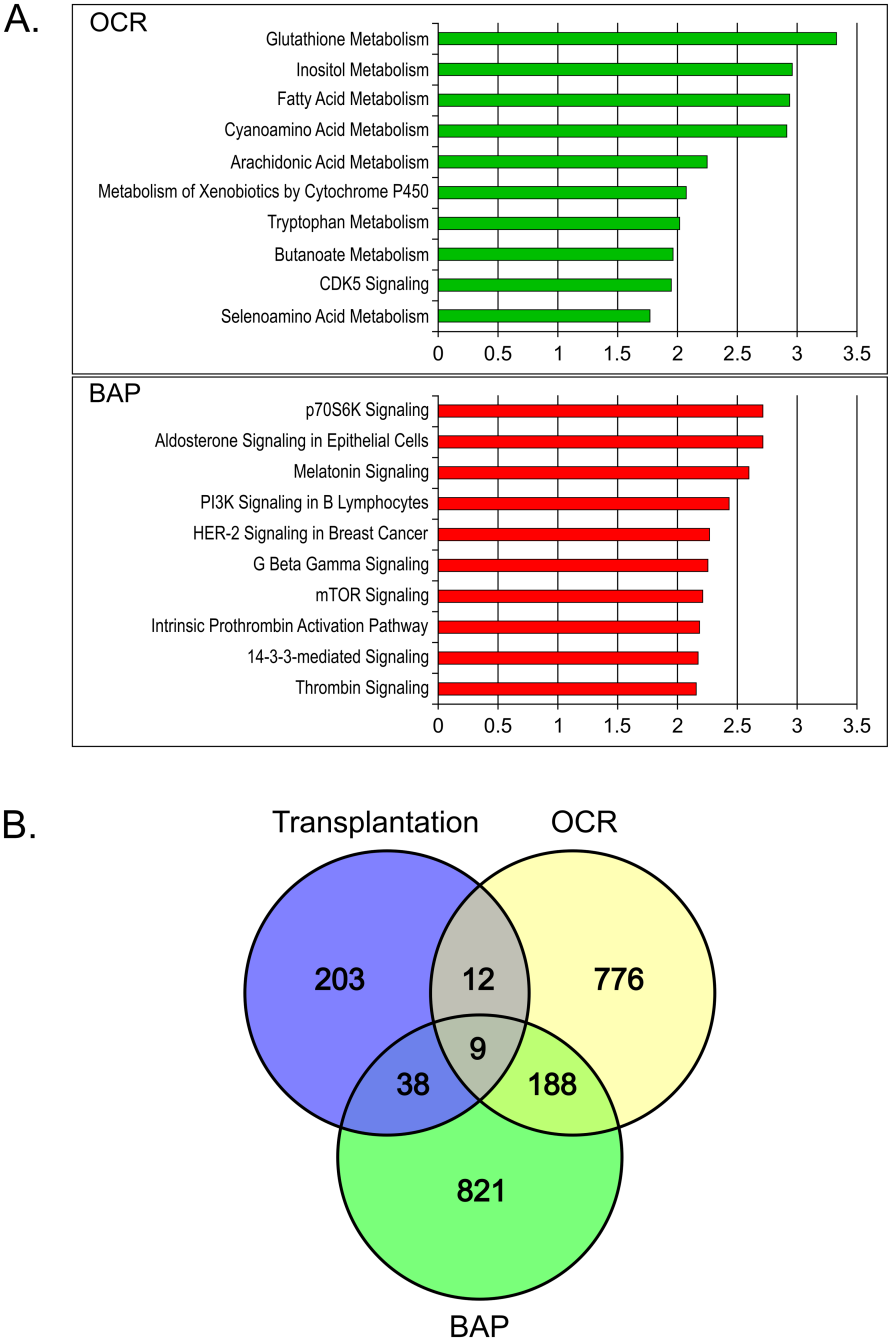
Canonical pathways of the OCR and BAP classifier sets. The results of Ingenuity Pathway Analysis of the probeset classifier lists associated with OCR and BAP. The top ten pathways associated with OCR (green) and BAP (red) are listed in order of the level of statistical significance (-log(p-value)). Pathways associated with OCR are mostly metabolic, whereas those associated with BAP are various signaling pathways. B. Venn diagram of the probeset classifier lists from diabetic mouse data (Transplant), OCR results, and BAP shows that there is overlap of only 9 probesets. This indicates that the three parameters are measuring distinct characteristics of islet function, which is supported by the diversity of functional pathways associated with each parameter. The diagram was created using the VENNY website tool [20].

The gene expression data were analyzed by class comparison based on these thresholds to define *good* and *bad* classes of islet preparations. This yielded a set of 985 probesets, representing 736 genes, that discriminated between high and low OCR, and a set of 1056 probesets, representing 790 genes that differed between high and low BAP. Pathway analysis revealed the OCR and BAP gene sets represented two strikingly different classes of genes functionally. *Good* islets, as defined by OCR, showed 16 significant pathways (p<0.005), a majority (65%) of which were associated with metabolism (Fig 4A). By contrast, the 44 significant pathways representative of *good* islets classified by BAP expressed greater association with signaling pathways, including key pathways related to islet biology, such as mTOR and AMPK signaling, and only two metabolic pathways (5%). These results suggest that OCR and BAP measured two distinct aspects of islet biology, as represented by transcriptional profiling. Moreover, comparison of the probeset classifiers derived from all three functional assays (reversal of diabetes in mice, OCR, and BAP) showed that the majority of genes associated with each metric was unique (Fig 4B).

### Development and verification of a diagnostic test for islet quality

Implementation of a “gene signature” for gauging islet quality within a clinical islet transplantation program requires a rapid, inexpensive, and reproducible method of measuring differential gene expression such as quantitative RT-PCR (qRT-PCR). To test this approach, an independent cohort of 16 new islet preparations comprising 8 *good* samples and 8 *bad* were analyzed by qRT-PCR for differential expression of a subset of the 36 probeset classifier for reversal of diabetes. The 36 classifiers represent 25 known genes, and of these there were twenty expression assays readily available on the qRT-PCR-based OpenArray TaqMan platform. These twenty genes represent 25 (69%) of the 36 probesets. The results show that expression of 10 of the 20 genes are significantly different (p<0.05) between islets that reverse diabetes and those that do not (Table 2). Analysis of these 10 significant genes as a single metric by two-way ANOVA proves highly significant (p<0.0001) for islet quality, and together have a predictive accuracy for reversal of diabetes of 86% (ROC analysis; Area Under the Curve = 0.8640 ± 0.0285). These results demonstrate that qRT-PCR can be a useful method for assessing expression levels of the gene classifiers and that the expression levels of this subset of 10 genes successfully predicts *in vivo* outcome in the diabetic mouse model.

**Table 2:** Analysis of a subset of genes by QRT-PCR.

Gene classifier	avg Good	avg Bad	Fold-differenceǂ	p-value
NOTCH2	0.480	1.087	2.27	0.00006
MAP3K5	0.325	0.618	1.90	0.00034
RND3	0.479	0.898	1.87	0.00526
CARD6	0.038	0.062	1.64	0.00791
ITGB6	0.148	0.337	2.27	0.00936
IFITM2	1.730	2.767	1.60	0.01448
KCNMA1	2.016	1.357	(1.48)	0.02373
MPZL2	0.257	0.428	1.66	0.02898
MYOF	0.521	0.963	1.85	0.03750
SEPT9	2.722	3.760	1.38	0.04863
PLSCR1	1.162	1.955	1.68	0.06242
FRMD4A	0.043	0.064	1.50	0.06996
PMEPA1	0.331	0.572	1.73	0.08153
DENND5B	0.866	1.132	(0.77)	0.10952
PTPN3	0.852	1.123	1.32	0.24673
MNX1	0.892	0.769	(1.16)	0.30835
MAPT	0.678	0.583	(1.16)	0.48698
PKIB	3.227	3.455	1.07	0.75409
TSHZ1	1.874	1.927	(0.97)	0.80347
ZC3H6	0.612	0.602	(1.02)	0.90909

^ǂ^ Ratio of expression in bad divided by good samples. Parentheses indicate the reverse ratio.

Comparing the results of the limited 14 probeset with those of the qRT-PCR revealed that 5 (KCNMA1, SEPT9, RND3, IFITM2, CARD6) out of 9 shared gene probes are among the 10 significant gene probes that can identify good from bad islets based on the qRT-PCR analysis. However, the other 5 gene probes (EST2, EST5, EHD4, MIR181A2, RNF187) in the 14 probeset were not among those included in the qRT-PCR study. Similarly, 3 (KCNMA1, RND3, CARD6) out of 4 shared gene probes in the further reduced 5 probeset were also among the same 10 significant qRT-PCR gene probes. One other shared probe had a p value of 0.08 (PMEPA1) in the qRT-PCR and the fifth (EST2) was not represented.

## Discussion

Transplantation of insulin-producing islet cells has been shown to be an effective treatment for severe type 1 diabetic patients. However, the effectiveness of the therapy varies greatly between islet transplantation centers [8,21]. It is widely accepted that this is predominantly due to the variability in islet preparation quality. And yet, effective and sensitive methods of gauging islet quality have been slow to develop. In this study, we hypothesized that the effectiveness of the islet graft depends both on beta cell function as well as the interaction between the graft and the host, and that these are governed by the expression of specific islet genes. Consequently, we examined the gene expression profiles of 59 human islet preparations using oligonucleotide arrays. 262 probesets, representing 199 individual genes, were identified that were differentially expressed between human islet preparations that were effective (*good*) or ineffective (*bad*) at reversing diabetes after transplantation in mice. The 262 probe classifier set predicted the ability of a specific preparation to reverse diabetes with 83% accuracy. A subset of 36 probesets had a similar predictive value, and 10 of the twenty-five genes represented in this subset were independently validated with a new set of samples by qRT-PCR.

A common theme from pathway analysis of the 262 classifier set was that a large number of significant classifiers were found to be associated with inflammation and other immune responses (Fig 3), some of which been reported to have roles in islet function and diabetes. For example, components of specific cytokine pathways are upregulated in *bad* islets, including tumor necrosis factor (TNF) machinery such as the TRAIL receptor TNFRSF10B, which is directly involved in T cell-induced beta cell death [22,23]. Also, both FAS and its ligand, FASL, which are associated with induction of beta cell apoptosis [24,25], are at higher levels in *bad* islets, suggesting that islet death-related pathways are already activated in these preparations even before transplantation. In addition to apoptosis, these pathways activate NFκB and AP-1 transcription factors, resulting in upregulation of inflammatory cytokine expression [26]. One of these, CCL2 (MCP1), is documented to promote a local proinflammatory environment associated with islet death and diabetes [16–19]. *Bad* islets also have a higher expression of the pattern recognition receptor TLR3, which is coupled to islet dysfunction and increased cytokine expression [27]. The elevated tissue factor (F3) expression is pro-inflammatory as well and inhibits islet graft function [16,28]. Other chemokine systems are also increased, such as TGFB2 and its receptor TGFBR1 and the IL13 receptor, OSMR, but these may initiate protective signals for islet cells [29–31]. Likewise, SERPINA3, also known as alpha-1-antichymotrypsin, is upregulated and in other systems is involved in wound healing [32,33]. So it appears that the pathways leading to islet dysfunction are already triggered before transplantation, but that there is also the initiation of some counteractive measures.

Conversely, a large number of genes that were preferentially upregulated in *good* islet preparations were associated with pancreas development and regeneration, suggesting that if repair/regeneration pathways were already initiated in damaged islets that they would be more likely to be effective after transplantation. Some of these genes include ONECUT1 (HNF6) [34,35], MNX1 (HB9) [36,37], NKX2-2 [38,39], INSM1 [40,41], NKX6-1 [38,42], FOXA2 [43–45], and PTCH1 [46,47] that interact in regulatory networks (Fig 3B) guiding embryonic pancreas development and regeneration following injury. Interestingly, another molecule implicated in this process, NOTCH2, is preferentially expressed in the *bad* islet preparations. A possible explanation lies in the importance of NOTCH2 in expansion of the progenitor cell population by suppression of neurogenin3-dependent endocrine cell differentiation [48–51].

To reduce the number of classifiers to a manageable level the 36 probeset list was subjected to logistic regression analysis with backward step-wise selection. The results indicate that the number of genes can be reduced to 14 or even 5 without loss of predictive power, though this of course must be evaluated experimentally. It is interesting to note, however, that the second highest scoring gene in this analysis was the rectifying potassium channel KCNMA1 which is upregulated in good islets and has been shown to be important for repolarization of the membrane following insulin secretion. Loss of KCNMA1 suppresses insulin secretion and increases susceptibility to oxidative stress and apoptosis [52]. Conversely, the sixth highest, SEPT9, is upregulated in bad islets and has recently been shown to be upregulated in islets of type 2 diabetics [53].

Due to the method by which the U133 Plus 2.0 GeneChips were developed, some of the best classifiers (6 of the 36 probeset list) were expressed sequence tags (ESTs) which were not mapped to coding regions. One of these, EST4, was subsequently mapped to microRNA MIR181A2, while the others appear to be in the 3’ untranslated regions of specific genes (EST1 in EGFR, EST2 in TRAPPC9, and EST3 in FOXE1). Two classifiers, EST5 and EST6, appear to be potential new genes, wojo and kyber respectively, of unknown function which were predicted by computational methods along with some expression evidence. One of these, wojo, was previously reported in a human islet cDNA screen (Melton et al, Endocrine Pancreas Consortium, unpublished).

The goal of the present study was to develop a diagnostic for assessment of the quality of cell preparations prior to use in clinical islet transplantation therapy for type 1 diabetes. After isolation, islets are typically infused into the patient within 24h-48h, and so methods of assessment must be rapid. For application of a “gene signature” of islet quality there are several methods for quantifying gene expression, and in this case we evaluated qRT-PCR analysis with a subset of 20 genes. Expression levels of half of these were significantly associated with reversal of diabetes in mice (Table 2) and together showed an 86% predictive accuracy for the outcome. Using the logistic regression model and step-wise simplification also suggest that use of as little as 5 gene probes (three of which are also represented in the 10 gene probes found significant in the qRT-PCR) could separate good from bad islet preparations with a maximum true positive predictive rate of >90% while maintaining a false positive rate of zero. Further studies will be required to determine whether this is the optimal set of classifiers for clinical application and whether qRT-PCR is the best method for utilization of this approach. For example, a limited evaluation of a bead-based RNA hybridization assay (Panomics Quantigene) with a small set of these genes provided similar discrimination between islet quality classes (data not shown). However, no matter the method of quantification it is our opinion that due to the heterogeneous nature of islet preparations that an effective diagnostic will require a set of genes and a strategy for combining the data into a meaningful metric. Logistic regression is one possible way to combine expression levels of several classifiers, such as in Fig 2 in which islet preparations that exceed a specified threshold would be considered transplantation quality.

We also investigated genes that correlated with two other standard measures of islet quality, namely glucose-responsive oxygen consumption rates (OCR) and beta cell apoptosis (BAP), in the hope of identifying one universal set of classifiers that could alone be used for preclinical islet assessment. However, it appears that each of these three parameters (reversal of diabetes in mice, OCR, and BAP) were correlated with different gene classifiers with overlap of only 9 probesets (Fig 4B). It is interesting that the genes associated with these different assays also reflect distinct biology with OCR associated with metabolism, BAP associated with various signaling pathways, and reversal of diabetes with inflammation and regeneration which require interactions within the organism. We are now of the opinion that all three of these islet assessments provide important complementary data for assessing islet function prior to transplantation.

A potentially confounding aspect of the current study is that islets contain several different cell types, each with unique gene expression profiles. This is especially true of human islet preparations, which have been shown to vary in the percentage of individual cell types by more than 300% [54]. Further complications are introduced by the effects of pancreas digestion and islet purification methods [55], as well as the islet restructuring that occurs during the unavoidable step of short-term post-isolation culture [56]. In the face of such complexity, we chose to implement an unbiased approach by profiling samples in parallel with transplantation into diabetic mice, i.e. without “islet picking”. We felt this would allow us to identify molecules that potentially affected engraftment in our islet recipients, without the bias of focusing on molecules from the insulin-producing beta cells. We are currently investigating the expression of several of the gene products in both human pancreata and the resultant islet preparations to identify the cell-type specificity of these molecules.

Another question which arises from this study is whether the gene classifiers were originally expressed in the donor organ or were expressed as a consequence of the islet isolation process. Islet isolation from human pancreata is a rigorous process involving both enzymatic and mechanical dissociation of the tissue followed by gradient separation of the cell clusters. It has been reported that this process, especially the gradient isolation, harms islets and makes them less suitable for cell therapy, although the specifics of the damage are still unknown [57]. Another recent study has shown significant changes in human islet gene expression in response to inflammatory cytokines [58]. We investigated our list of candidate molecules and found that some are present in the donor organ prior to processing and may correlate with certain islet characteristics; however, their ability to predict islet quality has yet to be determined.

An important aspect for the field of transplantation is the effective transfer of standardized diagnostic measures to other transplantation centers. This has been especially true in islet cell transplantation where the standard measures for islet assessment are known to be inadequate, but validation of new assays across centers has been difficult. We previously addressed this problem in the development of the glucose-responsive oxygen consumption assay which was validated in two centers simultaneously [15], but as far as we know this is the only new assay that has been compared in more than one center. By contrast, gene expression analysis is readily available at most research centers, and so the current approach to islet assessment may be evaluated at other centers.

In conclusion, our microarray-based analysis of 59 human islet preparations has identified a set of 262 probesets whose expression constitutes a “gene signature” of islet quality as it relates to cell therapy for diabetes. This gene set is being incorporated as part of the pre-transplant assessment of human islets for clinical transplantation therapy in our islet transplantation program. Further investigation of the role of these molecules in islet cell biology is ongoing. Moreover, the expression of the identified gene set is being determined in parallel to the OCR and BAP assays in prospective clinical trials in order to determine the relevance of each parameter and its influence on short and long term islet survival and function post-transplantation in humans.

## Materials and methods

### Human islet isolation and processing

Human islets were provided by the Southern California Islet Cell Resources Center (SC-ICRC) at City of Hope (Duarte, CA). The study was approved by the City of Hope Institutional Review Board and with the written informed consent from each organ donor for research use. Pancreata were digested by a modified Ricordi method [59] using Liberase-HI collagenase (Roche Molecular Biochemicals, Indianapolis, IN), then purified on a continuous Biocoll (Biochrom, Berlin, Germany) gradient in a cooled COBE 2991 Cell Processor (Gambro BCT, Lakewood, CO). Islet fractions collected from the COBE that had a purity >70% were pooled and cultured (1–2 days) in Miami Media #1 (Mediatech Inc., Herndon, VA) prior to RNA isolation and *in vitro* and *in vivo* analyses. Human islets were processed under strict GMP-compliant conditions, suitable for human clinical transplantation, using the same islet isolation protocol, facility and isolation team for each preparation. To obtain a true gene expression signature of the cell preparations used for islet cell therapy, human islet preparations were analyzed as they were received from the transplantation center, without manual selection of islets or other manipulations. Islet gene expression was analyzed using 59 individual human islet preparations. The average donor age was 44.0 ± 113.1 years (mean ± standard deviation; range 15–68 years) and 33 of the 59 (56%) pancreas donors were male. The average purity of the islet preparations was 73.6 ± 12.0% (mean ± standard deviation; range: 30–90%). Aliquots of these preparations were assessed (see below) for glucose-responsive oxygen consumption rates, beta cell apoptosis, and by transplantation into diabetic mice concomitant to RNA extraction to avoid potential bias in the gene expression introduced by differences in cell culture times.

### Measurement of glucose-responsive oxygen consumption rates (OCR)

The islet flow culture system has been described in detail previously [60]. Briefly, 750 unsorted cell clusters from each islet preparation were loaded in duplicate into the inverted perfusion system and absolute levels of OCR were calculated as the flow rate times the difference between inflow and outflow oxygen tension measured by the phosphorescence lifetime of an oxygen-sensitive dye that was painted inside the perifusion chamber [15]. Inflow oxygen tension remained constant during the course of the experiment [15], and was determined at the conclusion of each experiment after inhibiting cellular respiration by the addition of antimycin A [15]. The changes in OCR in response to glucose were calculated as the difference in OCR averaged from 30 to 45 min following the change to 20 mM glucose, and the 15 min prior to the change.

### Measurement of percent beta cell-apoptosis (BAP) by laser scanning cytometry

Laser scanning cytometry was performed as previously described [14]. Briefly, 500–1000 IEQ were fixed in 10% formalin, embedded in paraffin and sectioned at the City of Hope Anatomical Pathology Core or SC-ICRC facilities. Slides were immunostained for terminal deoxynucleotidyl transferase-mediated dUTP-biotin nick end labeling (TUNEL) using the ApopTag^®^ Plus Fluorescein *In Situ* Apoptosis Detection Kit (Millipore/Chemicon, Temecula, CA), following manufacturer recommendations, and for insulin using guinea pig anti-human insulin antibody as primary antibody (Linco Research/Millipore, St Charles, MO) and a Cy5 conjugated secondary antibody (Jackson Immuno-Research, West Grove, PA). Slides were scanned using a iCys laser scanning cytometer (40x objective, Compucyte, Woodbridge, MA. U.S.A.) and iCys 3.2.5 software. Cells staining for insulin were defined as beta cells; cells that co-stained for insulin and TUNEL were defined as apoptotic beta cells.

### Diabetes induction, islet transplantation and blood glucose monitoring

Mice were housed in specific pathogen free (SPF) conditions at the Animal Resources Center (ARC) of the Beckman Research Institute of City of Hope. NOD.SCID mice were obtained from the ARC Breeding colony at City of Hope, which were derived from breeder animals received from Jackson Laboratories (Bar Harbor, ME). The use of animals and the animal procedures were approved by the City of Hope Research Animal Care Committee. Diabetes was induced by intraperitoneal injection of streptozotocin (50 mg/kg, daily for 3 days, Sigma-Aldrich) freshly dissolved in citrate buffer. Blood samples were taken from the tail and glucose levels were measured using the One-Touch Ultra Blood Glucose Monitoring System (Lifescan Inc., Milpitas, CA). Animals were considered diabetic following two consecutive blood glucose measurements >400 mg/dL. The transplantation of 1000–2000 IEQ under the renal capsule of one kidney was performed as described previously [13,61]. Post transplant blood glucose measurements were taken two to three times per week. Islets were considered functional if the average blood glucose levels remained below 200 mg/dL, 3–4 weeks after transplantation. Islet preparations were tested in three or more animals; if the transplant successfully reversed diabetes for 3–4 weeks, the engrafted kidney was removed to ensure that glycemic reduction was dependent on the islet grafts. No reversal of diabetes was observed that was not graft-dependent.

### Gene expression profiling and analysis

RNA was extracted from islet preparations using Trizol (Invitrogen). Biotinylated cRNA was prepared using the Ambion MessageAmp Biotin II kit (Ambion) and hybridized to Affymetrix Human Genome U133 Plus 2.0 GeneChips which profiles the whole known human genome representing about 47,000 transcripts. Normalized signals were generated using quantile normalization (RMAExpress[62]). Batch effects were removed using ComBat [63] and the results were used for Class Comparisons (ANOVA) and Class Predictions (BRB Array Tools; http://linus.nci.nih.gov/BRB-ArrayTools.html). The 59 microarray datasets (data uploaded to GEO, GSE75062) were randomly assigned to two groups, the first was used to identify differentially expressed genes by Class Comparison and the second for testing the predictive power of each classifier by Class Prediction, and this randomization was repeated three times and the results pooled. Class predictions were performed using the Diagonal Linear Discriminant Analysis (DLDA) method, which is based on maximum likelihood discriminant rules that give consistently good results with our data set and others[64]. In addition to the above analysis, an independent analysis was done using the Partek Genomics Suite (Partek Inc.) to determine if the classifiers identified using BRB-Array Tools were reproducible. An ANOVA for differential expression was performed and the results compared to the genelists obtained using BRB-Array Tools. An identical approach to the BRB-Array Tools methodology was used to refine the gene signatures using 3 algorithms (DLDA, Random Forest and Linear Regression) in the Partek software. For analysis of genes associated with beta cell apoptosis and oxygen consumption rates the Class Comparison and Class Prediction each utilized the entire set of samples. Functional analysis was performed using Gene Ontology (GO) (http://www.geneontology.org/) and Ingenuity Pathway Analysis (IPA). Receiver Operating Characteristics (ROC) analysis was done using JROCFIT (http://www.rad.jhmi.edu/jeng/javarad/roc/JROCFITi.html). All the microarray data for this study are available for review at the NIH GEO accession site.

### QRT-PCR using OpenArray TaqMan arrays

Custom OpenArray plates were designed by comparing the available TaqMan assays from Applied Biosystems with the 36 gene classifier set obtained from the microarray analyses. The 36 Probesets represented 25 genes, and twenty of these had assays available (Table 2). Five reference genes were also chosen (HPRT1, GUSB, PPIB, ACTB and GAPDH) and post-analysis indicated that GUSB exhibited the most stable expression and so it was used for normalization of the results. Total RNA was isolated from sixteen new human islet preparations representing eight that reversed diabetes and eight that failed. The RNA samples were reverse transcribed using Superscript II (Life Technologies) and relative gene expression measured by PCR on an OpenArray NT using OpenArray Master Mix according to manufacturer instructions (Applied Biosystems). Assays were performed in duplicate for each sample on three separate days for a total of six technical replicates for each sample for each gene. The expression was quantified with the R program qpcR package[65] using the cm3 model[66]. Some of the sample wells (756 of 24192 total or 3.1%) failed amplification due to robot filling errors and the results were removed as outliers by ROUT analysis[67]. Expression values were normalized with the GUSB reference gene and technical replicates were averaged for each sample. The averages for each gene were used to assess significant differences in expression associated with islet quality.

### Statistical analysis

BRB ArrayTools and Partek Genomics Suite were used for analysis of gene expression data and the internal statistical procedures were utilized with alpha and beta set to 0.05 and fold-difference set to 1.5. Analysis of qRTPCR data was done using GraphPad Prism with a p-value < 0.05 considered significant. To narrow the focus of probesets that were found to successfully classify islets into good and bad preparations, the logistic regression analyses were performed using the microarray data. Treating islet quality indicator as the dependent variable, the predicted log odds of being classified as a good islet as opposed to being a bad islet are defined as scores. The probesets were individually screened for their ability to predict the genomic profile classification, their p-values were ranked and the maximum possible subset of the classifying probes were included in a multivariate model (full model). A backward step-wise model selection procedure was then used to reduce the number of classifiers. Boxplots and ROC curves (ROCR package [68]) were used to illustrate the classification performance of the selected models. Specifically, boxplots were used to demonstrate the separation between good and bad islet groups, and the ROC curves were used to evaluate the logit model in terms of the trade-off between true positive and false positive rates. The logistic regression analyses were performed using R statistical software (version 3.1.2).
